# Parental tuning of language input to autistic and nonspectrum children

**DOI:** 10.3389/fpsyg.2022.954983

**Published:** 2022-09-23

**Authors:** Angela Xiaoxue He, Rhiannon J. Luyster, Sudha Arunachalam

**Affiliations:** ^1^Department of English and Literature, Hong Kong Baptist University, Kowloon Tong, Hong Kong SAR, China; ^2^Department of Communication Sciences and Disorders, Emerson College, Boston, MA, United States; ^3^Department of Communicative Sciences and Disorders, New York University, New York, NY, United States

**Keywords:** parent, caregiver, language, receptive language, autism, processing, eye-tracking

## Abstract

Caregivers’ language input supports children’s language development, and it is often tuned to the child’s current level of skill. Evidence suggests that parental input is tuned to accommodate children’s expressive language levels, but accommodation to receptive language abilities is less understood. In particular, little is known about parental sensitivity to children’s abilities to process language in real time. Compared to nonspectrum children, children on the spectrum are slower to process language. In this study, we ask: Do parents of autistic children and those of nonspectrum children tune their language input to accommodate children’s different language processing abilities? Children with and without a diagnosis of autism (ages 2–6 years, *N* = 35) and their parents viewed a display of six images, one of which was the target. The parent labeled the target to direct the child’s attention to it. We first examined children’s language processing abilities by assessing their latencies to shift gaze to the labeled referent; from this, we found slower latencies in the autistic group than in the nonspectrum group, in line with previous findings. We then examined features of parents’ language and found that parents in both groups produced similar language, suggesting that parents may not adjust their language input according to children’s speed of language processing. This finding suggests that (1) capturing parental sensitivity to children’s receptive language, and specifically language processing, may enrich our models of individual differences in language input, and (2) future work should investigate if supporting caregivers in tuning their language use according to children’s language processing can improve children’s language outcomes.

## Introduction

Children learn the language or languages of their community by forming mental representations of the language they are exposed to. However, language input is not directly represented in children’s minds but is instead filtered through their own cognitive skills and linguistic knowledge. Their *intake* of the input is therefore limited by their own abilities (e.g., Trueswell and Gleitman, 2007; Kidd et al., 2013; Lidz and Gagliardi, 2014; Omaki and Lidz, 2015; He and Arunachalam, 2017; Arunachalam and Luyster, 2018; He, 2022). If, for example, parents produce language input that is too complex for the child’s current abilities, the child may not benefit from it (e.g., He, 2022). Therefore, to best support intake, input must be tuned to children’s language abilities.

There is evidence of this kind of tuning (also called accommodation) in caregivers’ language input. For instance, caregivers provide more complex linguistic input to children with larger expressive vocabularies or more advanced production of syntax than those with less advanced skills (e.g., Bernstein Ratner, 1984; Pan et al., 2005; Bornstein et al., 2007; Huttenlocher et al., 2010). Leung et al. (2021) found that parents adjust their way of speaking about animals based on their child’s lexical knowledge. They provide longer referential expressions (e.g., “the spotted yellow leopard”) if they report that the child does not yet produce the word than if they report that the child does produce the word (e.g., “the cat”). This prior research, however, has focused primarily on children’s *expressive* language levels. We know much less about whether caregivers’ language is tuned to children’s language *comprehension* abilities. This is an important gap to fill, for two reasons. First, children’s intake from the input is determined by their abilities to comprehend (not necessarily produce) the language they are exposed to. Second, language comprehension is harder to gauge in children; while parents know about children’s expressive skills from what they say, receptive skills can only be discerned indirectly, by observing behaviors such as whether children respond successfully to prompts (e.g., Tomasello and Mervis, 1994). If parents are less confident about what their child understands, they may have difficulty tuning their language input to their child’s language comprehension abilities.

One recent study looked at parent tuning to children’s receptive language: Arunachalam (2016) found that children processed noun phrases with postnominal modifiers (e.g., an umbrella with stripes) more quickly than those with prenominal modifiers (e.g., a striped umbrella), and correspondingly, parents more often labelled objects with postnominal modifiers, especially when the task was harder. This suggests that parents were attuned to what their child would find easier and more difficult to process and adjusted their language accordingly. The current study is a replication and extension of Arunachalam’s (2016) work, and we return to and describe it in more detail below. For the moment, we address why language *processing* is particularly important to consider in this context.

By language processing, we are referring to real-time language comprehension. This requires the child to access representations for the words they hear, build a syntactic parse, and integrate this information with real-world knowledge about what the speaker is likely to be speaking about. Language processing speed is typically measured by showing the child two pictures and labelling one of them (e.g., “where’s the cat?”); the child’s latency to look to the cat is taken as a measure of how quickly they have processed the auditory label and identified the correct referent (e.g., Fernald et al., 2008b). Children who are faster to process the language they hear have larger vocabularies (e.g., Fernald et al., 2006; Weisleder and Fernald, 2013) and have more opportunities to learn new words (e.g., Fernald et al., 2008a; He et al., 2020).

For some children, real-time language processing is a particularly difficult task. Children on the autism spectrum, for example, show slower language processing than their nonspectrum peers, both when the groups are matched on chronological age (e.g., Bavin et al., 2014; Bavin and Baker, 2017) and when they are matched on language level on standard assessments (e.g., Ellis Weismer et al., 2016; Hartley et al., 2020). This suggests that language processing may be particularly affected in autism above and beyond aspects of language that are measured on standard assessments, such as vocabulary size. Therefore, it may be especially important for autistic^1^ children that their caregivers’ language is tuned to their language processing abilities (e.g., Adamson et al., 2009; Bottema-Beutel et al., 2014; Yoder et al., 2015; Fusaroli et al., 2019). Perspectives from autistic adults are valuable for understanding the autistic experience of linguistic processing demands; one blogger writes on her blog “Musings of an Aspie”: “I have all sorts of communication glitches. I struggle with verbal instructions. If there’s background noise or other distractions, my auditory processing lags to the point that it can take a few seconds to process speech from noise into words” (Kim, 2013).

In the current study, we ask whether parents of autistic children, like the parents of nonspectrum children in Arunachalam (2016), tune their language input by producing language that is easier to process. We chose this population for two additional reasons. First, some autistic children have relatively more impaired receptive language than expressive language (e.g., Artis and Arunachalam, submitted; Charman et al., 2003; Luyster et al., 2007; Ellis Weismer et al., 2010; but see Kwok et al., 2015). Second, just as language comprehension is difficult to measure in nonspectrum children because it relies on their response to prompts, the difficulty is amplified in autistic children, who are likely to show differences in social reciprocity and responsiveness (APA, 2013). Because of both of these factors, parents of autistic children may find it especially difficult to gauge—and tune to—their child’s language comprehension and processing abilities.

Indeed, we are not aware of any previous studies examining how parent language input is tuned to autistic children’s language comprehension or language processing specifically. But there is an ample literature comparing caregiver language input provided to autistic children and input provided to nonspectrum children, which offers a relevant backdrop. In general, many of these studies report group similarities in the input: parents of autistic children use similar language as parents of nonspectrum children with respect to broad measures such as MLU, word tokens, word types, and lexical diversity (e.g., Swensen, 2007; Swensen et al., 2007b; Warren et al., 2010; Bang and Nadig, 2015; Nadig and Bang, 2017; Fusaroli et al., 2019; see Bang et al., 2019 for a review), at least when the groups are matched on expressive language level. Even with infants who have not (or yet) received an autism diagnosis but are at either higher or lower likelihood of receiving such a diagnosis based on whether they have an older autistic sibling, children in both groups receive a similar amount of infant-directed speech (see Woolard et al., 2021 for a recent scoping review) and this input is similar in features such as number of word tokens and types (although by 18 months, infants with higher autism likelihood hear language with a lower MLU; Choi et al., 2020).

These findings suggest a puzzle. Autistic children, who often have a different developmental profile (e.g., slower processing speed) may require *different* kinds of language input for optimal intake, and given parents’ sensitivity to children’s language abilities, we might predict that parents would therefore provide different kinds of input. Indeed, when more specific parent language features are studied, group differences do appear. For example, parental input to autistic children (compared to that to nonspectrum children) contains fewer questions (e.g., Venuti et al., 2012; Goodwin et al., 2015; Luyster et al., 2022), fewer comments related to story characters’ mental states (Slaughter et al., 2007), and more utterances differing in pragmatic appropriateness (Landa et al., 1992; Losh et al., 2008; Stern et al., 2017). Moreover, because first-degree relatives of autistic individuals are more likely to have traits in the broader autism phenotype than the general population, some parents of autistic children also show some traits associated with autism that differ from parents of nonspectrum children. In particular, some of these parents use a slower speech rate and have prosodic characteristics associated with autism (e.g., Patel et al., 2020).

Some of these features of parent speech could facilitate language comprehension in autistic children. For example, differences in play behavior and responsiveness in autistic children may mean that some kinds of parent interaction and parent language input are more effective than they are for nonspectrum children (e.g., Haebig et al., 2013; Bottema-Beutel et al., 2014). Bani Hani et al. (2013) found that when parents introduced novel words to their child, parents of children on the spectrum used multiple nonverbal cues (e.g., eye gaze, pointing) accompanying the new word, perhaps in order to maintain the child’s attention given their knowledge of attentional differences in autistic children (generally) or their child (specifically). With infants, the review paper mentioned above (Woolard et al., 2021) also reported evidence of group differences in subtle behaviors––parents of higher-likelihood and later-diagnosed children produce infant-directed speech with more attention bids and more follow-in commenting. They also use the infant’s name more often (He et al., 2018) and produce more gestures (Talbott et al., 2015). All of these behaviors may help the parent get and maintain the child’s attention, the importance of which has been noted elsewhere for both naturalistic and clinical settings (Constain et al., 2018). Thus, previous findings are consistent with the hypothesis that parents of autistic children are sensitive to their child’s attentional skills and tune their input accordingly.

However, we do not yet know the extent to which parental language input is specifically tuned to children’s language processing abilities. Slower processing speed in children on the spectrum (e.g., Bavin et al., 2014; Ellis Weismer et al., 2016; Bavin and Baker, 2017; Hartley et al., 2020; Horvath and Arunachalam, under revision) may mean that the best input for them is slower and/or consists of easier-to-process constructions (e.g., active instead of passive, e.g., Abbot-Smith et al., 2017 and Messenger et al., 2011; or postnominal modifiers instead of prenominal modifiers, as we investigate in the current study, e.g., Sekerina and Trueswell, 2012).

To summarize, past work suggests that there are both similarities and differences in the parental language input directed to autistic vs. non-spectrum children. However, one remaining gap that we think is particularly important is whether (and if so, how) the input might be tuned to children’s real-time language processing.

In the current study, we ask whether parents’ language input is tuned to autistic children’s real-time language processing abilities by replicating and extending a study with nonspectrum children by Arunachalam (2016). In that study, parent–child dyads played a finding game. On each trial of the game, an array of six pictures was displayed on an eye-tracking monitor. The parent was directed to describe one of them so that their child could identify it. Parents were not told what to say, only which picture they should talk about. Arunachalam examined both children’s speed of looking to the target—their language processing speed, a real-time index of language comprehension—and features of parental language input. Task difficulty was manipulated across two conditions^2^. In the Hard condition, the target object had a competitor in the display from the same basic-level category that differed in some salient property (e.g., two books: one open, one closed). In the Easy condition, there were no competitor objects from the same basic-level category. In the Hard condition, parents would have to use a more complex referential expression to label the object (e.g., “the open book” or “the book that’s open”); in the Easy condition, although parents were still free to use those complex expressions, the additional modifiers would be unnecessary for target identification.

A large psycholinguistics literature establishes that young children can process noun phrases incrementally and can correctly interpret modifiers as disambiguating information when multiple objects from the same category are present. This holds not only for nonspectrum children (e.g., Thorpe and Fernald, 2006; Fernald et al., 2010; Huang and Snedeker, 2013; Davies et al., 2021), but also for children on the spectrum (e.g., Bavin et al., 2016; Bavin and Baker, 2017). Thus, Arunachalam’s (2016) method applies well to autistic children. In the current study, we therefore replicate and extend this study, focusing on autism, with nonspectrum children as a comparison.

With respect to children’s processing, Arunachalam (2016) found that children had shorter latencies to look to the target, that is, they were faster to process the parent’s referential expression, in the Easy condition than in the Hard condition. In the current study, we predict the same to be true for both the nonspectrum and autistic groups. Further, we expect children in the autistic group to be slower overall than those in the nonspectrum group, reflecting the slower language processing speeds found throughout the literature.

With respect to parental input, Arunachalam (2016) examined two main features: speech rate and type of referential expression. Because a slower speech rate can better support children’s language comprehension, particularly in difficult tasks (e.g., Haake et al., 2014), she expected a between-condition difference––slower speech rate in the Hard condition than the Easy condition. But this hypothesis was not borne out. However, in the current study with two groups of children––autistic and nonspectrum, given their differences in processing speeds, there might still be a between-group difference (despite a potential lack of between-condition difference)––specifically, it might be the case that parents of children on the spectrum would use a slower rate (than parents of nonspectrum children) in order to accommodate their slower processing speeds.

With respect to type of referential expression, Arunachalam (2016) coded whether parents labeled the target object with just a content noun (e.g., “the book”) or whether they added modifiers, and if the latter, whether the modifiers appeared before the noun (e.g., “the open book”) or after it (e.g., “the book that’s open”). Prenominal modifiers have been shown to be difficult for children to process (e.g., Sekerina and Trueswell, 2012; Huang and Snedeker, 2013; Arunachalam, 2016; but see Davies et al., 2021) and so postnominal modifiers should be preferable. What Arunachalam (2016) found was that parents did produce more postnominal modifiers, but only in the Hard condition—when the child’s task was more difficult, parents alleviated the difficulty by producing an easier-to-process referential expression. In the Easy condition, parents appeared less concerned about processing difficulty; even though modifiers were unnecessary to uniquely identify the referent (e.g., there was only one book in the display), parents did sometimes produce modifiers, and half of these were prenominal as compared to postnominal. This is interesting given that unnecessary modifiers increase processing load, even in adults (e.g., Engelhardt et al., 2006, 2011) as well as in children (e.g., He et al., 2020). Based on these findings, for the current study, we predict that parents will use more postnominal modifiers in the Hard condition than the Easy condition. This between-condition difference might be larger for the autistic group than the nonspectrum group, due to autistic children’s slower processing. Further, given that unnecessary modifiers also place an additional processing burden on the child, we predict that parents of autistic children will produce fewer unnecessary modifiers (i.e., fewer modifiers in the Easy condition) than parents of nonspectrum children.

Finally, we included exploratory analyses relating children’s processing to features of parents’ referential expressions. These analyses are exploratory because the number of data points of each type is unequal, and determined by parents’ referential choices. However, the findings provide hypotheses to test in future controlled experiments. Specifically, we examine whether children’s latencies to look to the target are predicted by the parents’ choice to include unnecessary modifiers in the Easy condition, as well as whether they are predicted by modifier position (prenominal or postnominal) in either condition.

To summarize, our goal in the present study was to replicate Arunachalam’s (2016) work with nonspectrum children and their parents, and to extend it to autistic children and their parents. Our overarching hypotheses were that we would replicate prior findings that autistic children are slower to process language than their nonspectrum counterparts, and that their parents would tune to this difference in processing speed by producing easier-to-process language: slower, with fewer modifiers, and with postnominal rather than prenominal modifiers.

Like Arunachalam (2016), we focused on young children (i.e., preschool and early school-aged), who are old enough to understand the task. Several studies have assessed online language processing in autistic children or children with an older autistic sibling in this age group (e.g., Swensen et al., 2007a; Venker et al., 2013; Brady et al., 2014; Chita-Tegmark et al., 2015; Horvath et al., 2018; Zhou et al., 2019). Further, this age group is optimal for studying language input and intake in parent–child dyads because these children are young enough that parents are still an important source of language input but old enough that parents have had ample time to observe their child’s language growth and evaluate their expressive and receptive language skills.

## Materials and methods

### Participants

Nonspectrum and autistic children participated with one of their parents. Participants were primarily recruited from the greater Boston area in the United States using online advertisements and our lab’s databases of families who expressed interest in participating in research. Some children in the autistic group were recruited through the Simons Foundation Powering Autism Research for Knowledge (SPARK) database (SPARK Consortium, 2018). All recruitment and testing procedures were approved by Boston University’s Institutional Review Board. A total of 20 autistic children (3 female, 17 male) and 15 nonspectrum children (8 female, 7 male) were included in the final sample. In each group, three of the participating parents were male; the rest were female. Six additional children participated in at least some elements of the study protocol but were excluded from the final sample: 4 had been assigned to the autistic group based on parent report of an autism diagnosis but failed to meet diagnostic criteria during the study (see below); 1 had been assigned to the nonspectrum group but scored above the autism threshold on the Social Communication Questionnaire (SCQ, see below); and 1 (in the autistic group) was unwilling to complete the experimental task.

The autistic group’s mean age was 4:9 (range 3:6 to 6:10) and the nonspectrum group’s mean age was 3:6 (range 2:1 to 4:5). We intentionally recruited nonspectrum children at younger ages to yield two groups that did not significantly differ on language or cognitive ability (see below). In both groups, dyads were included if parents reported that children were English learners with no more than 30% exposure to another language and had no known developmental disorders aside from either autism (for the autistic group) or those that are often comorbid with autism such as ADHD.

For the autistic group, diagnosis was confirmed using the Autism Diagnostic Observation Schedule-2^nd^ Edition (ADOS-2; Lord et al., 2012), the gold standard diagnostic instrument for autism spectrum disorder, by a research-reliable examiner. The ADOS-2 is appropriate for children with a chronological and developmental age of at least 12 months through adults. None of the participating parents nor other adults in their household self-reported as being on the spectrum.

For the nonspectrum group, we used the SCQ (Rutter et al., 2003) to confirm *via* parent report that the child was not exhibiting features indicative of autism. This questionnaire is normed for children 48 months and older, but it has been widely used with younger children (e.g., Marvin et al., 2017). Following Corsello et al. (2007), we used a threshold of ≥15 for children 48 months (and older) and a downward adjustment to ≥12 for younger children. All 15 nonspectrum children included in the final analyses scored below the relevant cutoff. None of the nonspectrum children were reported to have a household member with autism.

To obtain a picture of children’s language and developmental profiles and to ensure that the two groups did not significantly differ from each other, we asked parents to complete the MacArthur-Bates Communicative Development Inventory II Short Form A: Words and Sentences for expressive vocabulary (Fenson et al., 2007; three nonspectrum children did not have MCDI scores). This form is designed for typically-developing children ages 16–30 months; however, the publishers note that it “may be used with older, developmentally-delayed children” (CDI Advisory Board, n.d.) and many studies do so (e.g., Hambly and Fombonne, 2012; Robertson et al., 2017; Arunachalam et al., 2022). We also note that neither group was at ceiling (see Table 1). Most children also completed the Visual Reception, Receptive Language, and Expressive Language subscales of the Mullen Scales of Early Learning (MSEL; Mullen, 1995; due to scheduling difficulties, 1 autistic child and 4 nonspectrum children did not complete the MSEL). The MSEL is designed for children from birth to 68 months. The Visual Reception subscale serves as a rough proxy for nonverbal cognition, while the Receptive and Expressive Language scales serve as an additional measure of language level. *T-*tests showed no between-group differences on any of the scores: MCDI expressive vocabulary, MSEL Visual Reception, MSEL Receptive Language, MSEL Expressive Language (all *p*s greater than 0.1). See Table 1. Unsurprisingly given the heterogeneity of the autistic population, the standard deviations were larger for the autistic group than the nonspectrum group. Some of the children also participated in an unrelated experimental task (Clancy et al., 2019).

**Table 1:** Children’s language and cognition scores on standard assessments.

	*N*	Age, months mean (SD)	MB-CDI 2 mean (SD)	Mullen VR raw score mean (SD)	Mullen RL raw score mean (SD)	Mullen EL raw score mean (SD)	SCQ mean (SD)
Nonspectrum	15	41.93 (6.95)	87.18 (19.83)	41.60 (6.31)	40.18 (6.03)	40.64 (5.48)	4.91 (3.30)
Autism	20	57.85 (10.21)	69.30 (32.69)	37.33 (11.81)	35.44 (9.85)	35.11 (10.10)	14.28 (6.39)

MB-CDI 2, MacArthur-Bates Communicative Development Inventory Words and Sentences Short Form A (total number of words, out of 100, reported to be in the child’s expressive vocabulary); Mullen VR/RL/EL, Mullen Scales of Early Learning Visual Reception, Receptive Language, Expressive Language subscales. There were no significant differences between the two groups on these measures.

### Materials and apparatus

**Figure 1:**
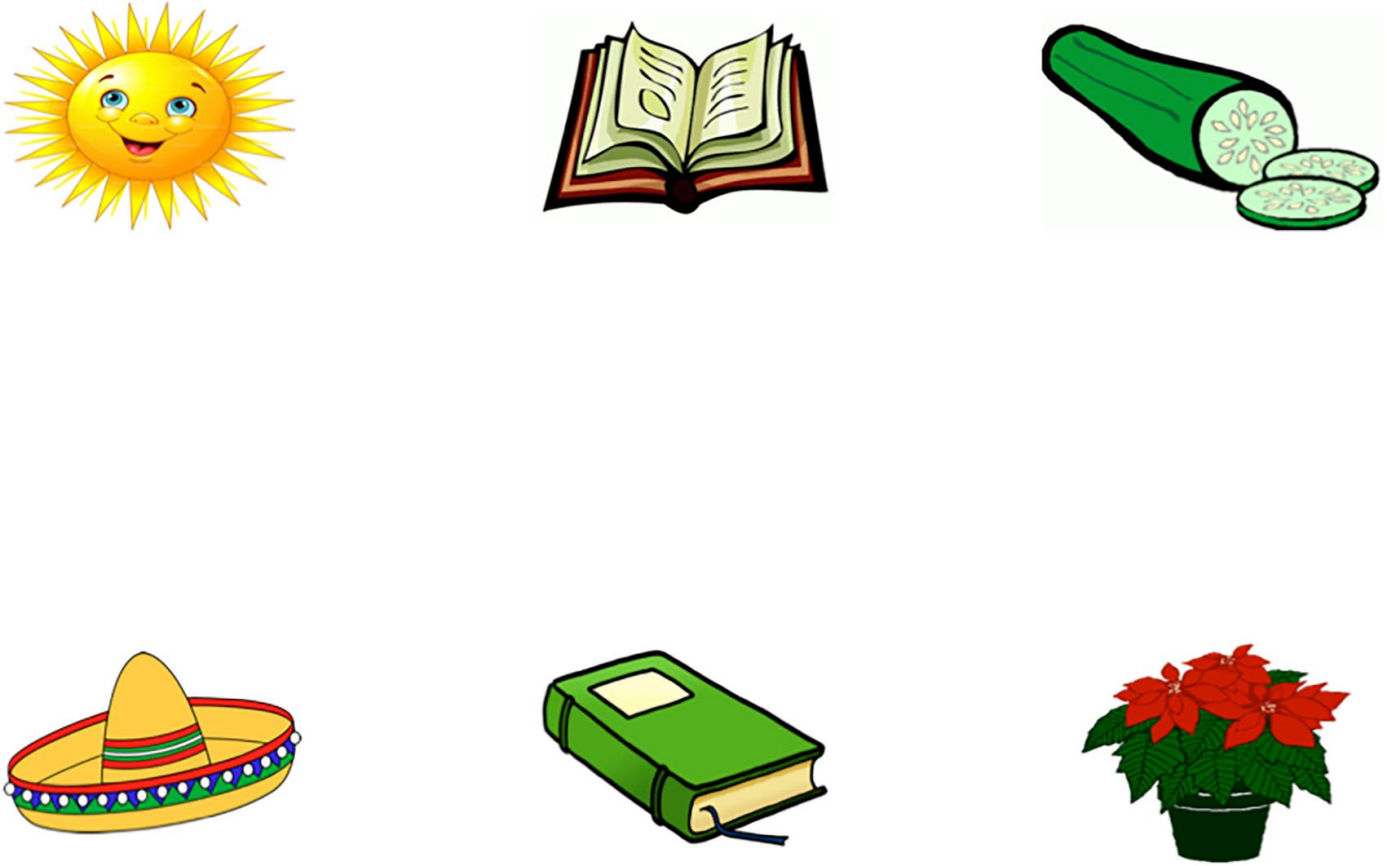
Sample trial in the Hard condition. The target image was the open book, top row middle.

The experimental task had 16 trials and 2 conditions, both within-subjects. On each trial, an array of 6 images arranged in 2 rows of 3 was shown (see Figure 1). Each image was contained in an invisible square measuring 570 pixels (px) × 410 px, with 65 px of white space between the columns and 255 px of white space between the rows. The images were similar to those used by Arunachalam (2016). To control somewhat for perceptual and conceptual complexity, we used clip-art images of highly familiar objects, animals, and people. In pilot work for this study, these images elicited referential expressions from parents that were similar to those in Arunachalam (2016) and that successfully and uniquely identified the target referent.

The two conditions, Hard and Easy, each had 8 trials. In the Hard condition, trials were characterized by having one pair of images from the same basic-level category but differing in some salient property (e.g., two stars, one red and one blue); one of these objects was the target. In the Easy condition, trials had no distractor objects from the same basic-level category as the target, and there were no pairs from the same basic-level category within the distractors. See Figure 1 for a sample trial.

All of the target images depicted common household objects, people, and animals; see Supplementary Materials for a full list. The properties on which the two competitor objects differed included color (e.g., red, blue) and size (e.g., big, small) as well as other properties that were easily discernible from the images (e.g., open, closed; asleep, awake; spotted, striped). Although parents were free to use any kind of modifiers they wished to describe the objects, the most salient property differences were describable by adjectives that children at this age would know. These included adjectives denoting color and size concepts (e.g., red, big). Note that particular modifiers may differ in whether they are more likely to be used prenominally than postnominally (e.g., color adjectives almost universally appear prenominally in English; Thorpe and Fernald, 2006). However, because our goal is to investigate differences between the Hard and Easy conditions, we were not concerned about potential differences at the level of individual modifiers.

The stimuli were presented on a Tobii T60XL eye-tracker sampling at 60 Hz, operating Tobii Studio software. Children sat in a car seat 20 in. in front of the eye-tracking monitor. The parent sat next to the child, approximately 3 feet away, and wore laser safety glasses that blocked the near-infrared wavelengths used to detect gaze but not shorter wavelengths. The parent could therefore see the screen, but their own gaze was not tracked.

### Procedure

The visit began with children playing with toys and parents completing paperwork, including providing informed consent on behalf of themselves and their child. The instructions were administered as in Arunachalam (2016). First, the experimenter explained to the parent that the dyad would see six images on the computer screen and the parent’s job was to get the child to identify the target image as quickly as possible. The parent was told they would need to describe the target image so that their child could identify it. The parent was instructed that they could say whatever they wanted to encourage their child to identify the correct image, but that because it was a guessing game, they could not point or use their hands. We explained that each of the 6 possible image locations was numbered, and for each trial, we would indicate to the parent which image was the target on each trial by referring to its numbered location.

Then, the parent and child entered the testing room, where the child was seated in front of the eye-tracker and the parent next to the child. The child first underwent a 5-point calibration procedure using Tobii Studio software. Before each trial, the experimenter, who sat behind and to the side of the parent, out of view of the child, held up a card depicting six numbers arranged in a two-by-three grid (from left to right: top row 1, 2, 3; bottom row 4, 5, 6). The same grid appeared on the computer screen, but displaying images instead of numbers, with each image on the screen corresponding to one number on the card. When the experimenter pointed to a number on the card, the parent thus knew which image was the target image on that trial, but the child, who could not see the card with the numbers, did not know. Therefore, parents were not told what to say, only which picture they should talk about.

On each trial, the experimenter operating the eye-tracking software from behind a curtain advanced the display so that the array of images was shown. The experimenter waited approximately 5 s to allow both the parent and child to examine the images and then showed the parent a new card with the target image’s number, after which the parent described the image that corresponded to the number. Children were not required to point to the image, as we were concerned that some autistic children might not point (and several in fact did not); if they did not point, the experimenter waited approximately 10 s or until the parent asked to move to the next trial. The duration of the task differed depending on how much or little the parent said, but the average duration was 7 min 46 s (*sd* = 163 s) for the autistic group and 6 min 46 s (*sd* = 120 s) in the nonspectrum group.

After the experimental task, most children completed the subscales of the MSEL with a trained researcher. Children in the autistic group only were administered the ADOS-2 to confirm autism diagnosis on a second visit to the lab, approximately 1 week later.

### Coding and analysis

Children’s processing and parents’ language were coded and analyzed as follows.

*Children’s processing.* Following past work in the language processing literature with both children on the spectrum and nonspectrum children (e.g., Fernald et al., 2008a; Venker et al., 2013), we used latency to look to the target as an index of processing speed. That is, we measured how quickly children shifted their eye gaze toward the target image from the offset of the referential expression produced by the parent––for instance, upon hearing “Look at the bear who’s sleeping,” how much time elapsed before the child’s first look toward the image of the sleeping bear. Following Arunachalam (2016), latencies were calculated from the offset of the referential expression, but negative latencies (i.e., looks after the onset but before the offset of the expression) were included (only one negative latency occurred in the final data set). (See more about referential expression coding below.) A look was defined as three consecutive frames for which the child’s gaze fell within the target area of interest (i.e., one of the six invisible squares); the first of these frames was used to calculate latency.

For gaze analysis, we excluded children and trials with excessive track loss (i.e., sampled frames without gaze coordinates, due to blinks or excessive movement). We first excluded children with 65% or more track loss across the entire experiment (4 children from the autistic group) and then excluded individual trials from the remaining children with 65% or more track loss (autistic, 48 trials; nonspectrum, 11 trials). We further excluded trials on which children did not look at the target at all before the trial ended (autistic, 8 trials; nonspectrum, 13 trials). Finally, we excluded from this analysis trials on which parents’ referential expressions were not codable, as discussed below. Therefore, the final sample for eye gaze analyses included 203 trials from 16 autistic children and 194 trials from 14 nonspectrum children.

**Figure 2:**
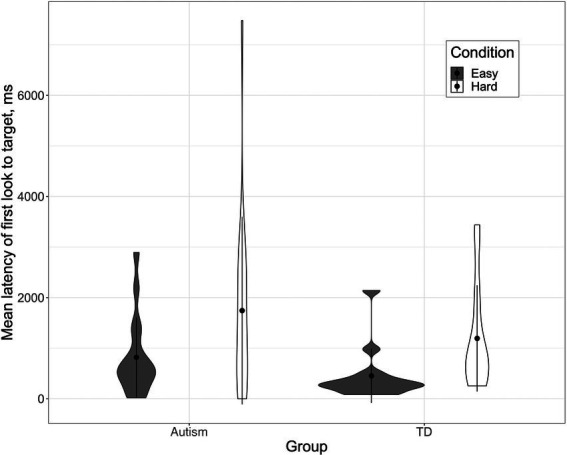
Violin plot depicting mean latency by participant of first look to the target, in ms, by group and condition. The black dot indicates the mean, and the shape indicates the probability density of the data at different values.

To analyze children’s gaze, we conducted linear mixed-effects regressions with the lme4 package version 1.1.28 (Bates et al., 2015) in R version 4.1.2 (R Core Team, 2016). The lmerTest package version 3.1.3 (Kuznetsova et al., 2017) provided *p*-values, using *t*-tests fit by Satterthwaite’s method. Pairwise comparisons of estimated marginal means were used to examine significant interactions using the emmeans package version 1.7.4–1 (Lenth, 2022). Figure 2 was made using the ggplot2 package (Wickham, 2009). The goal of these analyses was to understand whether autistic children were slower to process their parents’ referential expressions than nonspectrum children, whether children were slower in the Hard condition than the Easy condition, and whether there was an interaction between group and condition such that autistic children had particularly long latencies in the Hard condition. Because groups differed significantly on chronological age (although they did not differ significantly on language or cognitive measures), we included age as a fixed factor in the analyses.

*Parents’ input.* For analyses of parental input, parents’ speech was first transcribed by a trained experimenter and coded by two trained coders using Praat (Boersma and Weenink, 2020). Then, *speaking rate* and *referential expression choices* were analyzed. Speaking rate was calculated by dividing the referential expression duration (in seconds) by the number of syllables it contained. Referential expression durations were the period of time from the onset and offset of the referential expression. For referential expression choices, we specifically coded whether a referential expression was modified or not––in particular, to what extent unnecessary modification (i.e., overmodification) was used; and for modified, where the modifiers were positioned (i.e., before or after the noun).

Referential expressions were defined as a noun plus any modifiers from any syntactic category (excluding determiners, because preliminary coding showed that it was difficult to code their onset reliably given their brief duration). Examples of referential expressions produced by parents included: “little piano,” “book that’s open,” “doll in a pink dress,” “green hat with green dots on it.” Note that we included modifying information as part of the referential expression whether or not it was critical for identifying the target (that is, the offset of entire phrase, “green hat with green dots on it,” was used even if there was only one hat in the display).

For this analysis, we excluded trials on which parents produced referential expressions that could not be coded (31 trials in the autistic group, 24 in the nonspectrum group). These were trials on which the parent made a reference that was specific to their family (e.g., “Which one is Aunt Debbie’s favorite?”), trials on which the parent spoke about the incorrect target image, and trials in the Hard condition on which the parent did not provide sufficient disambiguating information to uniquely identify the target (e.g., saying “Where’s the book?” when there were two books in the array). One parent in the nonspectrum group produced only referential expressions of the family-specific type and this dyad was therefore excluded from all analyses. The final sample for parent speech analyses included 278 trials from all 20 parents of autistic children and 202 trials from 14 parents of nonspectrum children.

As with children’s gaze, we used linear mixed-effects regression to understand parents’ referential expression choices. We also used this approach in exploratory analyses to look for relations between children’s processing and parents’ referential expression choices. Models are specified in detail in the Results section below.

## Results

### Children’s processing

We ran a linear mixed-effects model with latency as the dependent measure; child age (centered around its mean), group (autistic vs. nonspectrum, contrast coded with autistic as 0.5 and nonspectrum as −0.5), condition (Hard vs. Easy, contrast coded with the Easy condition as −0.5 and the Hard condition as 0.5), and the interaction between group and condition as fixed factors; and participant and trial as random factors. This analysis yielded no significant effect of age (*β* = −19.58, *p* = 0.27), but it did reveal significant main effects of group (*β* = 790.86, *p* = 0.04) and condition (*β* = 750.22, *p* = 0.04). The interaction between group and condition was not significant (*β* = 149.76, *p* = 0.74).

These results indicate, first, that autistic children’s latency to identify the target (*m* = 1,327 ms*, sd* = 2,755 ms) was significantly slower than that of nonspectrum children (*m* = 796 ms*, sd* = 1895 ms). Thus, as predicted, autistic children were slower to process their parent’s speech. Second, latencies were longer in the Hard condition (*m* = 1,459 ms, *sd* = 3,084 ms) than the Easy condition (*m* = 686 ms, *sd* = 1,303 ms). Thus, also as predicted, children across both groups showed more difficulty in identifying the target when there was a competitor object than when there was not.

### Parents’ input

Parents’ speaking rate and referential expression choices were analyzed.

*Speaking rate.* Across all trials, mean speaking rate was 0.39 syllables per second for parents of autistic children (*sd* = 0.77) and 0.33 for parents of nonspectrum children (*sd* = 0.20). A linear mixed-effects model with speaking rate as the dependent measure, participant and trial as random factors, and group (autistic vs. nonspectrum) as a fixed factor (contrast coded as described above for child gaze analyses) revealed no main effect of group (*ß* = 0.068, *p* = 0.27).

*Referential expression choices––Overmodification.* In the Easy condition, where modifiers were not needed in order to specifically identify the target, parents nevertheless often produced unnecessary modifiers (replicating Arunachalam, 2016). For the autistic group, parents did so on 51% of trials, and for the nonspectrum group, 60%. Although this numerical difference between the groups was in the predicted direction (that is, we expected that parents of autistic children to more actively avoid overmodification in order to reduce the child’s processing burden on the child), it was not statistically significant (Fisher’s exact test *p* = 0.20).

*Referential expression choices––Modifier position.* Recall that we predicted that parents would use more postnominal modifiers in the Hard condition than the Easy condition, and that this between-condition difference might further be larger for the autistic group. The proportion of modifiers that were postnominal as compared to prenominal (excluding trials on which no modifiers were produced or on which both pre- and postnominal modifiers were produced) for each condition and group is shown in Table 2. As predicted, more postnominal modifiers were produced in the Hard than the Easy condition in both groups, and Fisher’s exact tests demonstrate that the difference in pre- vs. postnominal modifiers between conditions is significant for the autistic group (*p =* 0.008) but not for the nonspectrum group (*p* = 0.3). Thus, the results suggest that parents of autistic children may be particularly sensitive to the difficulty of the Hard condition as compared to the Easy condition.

**Table 2:** Proportion of modifiers that were postnominal as compared to prenominal produced by parents by condition and group (trials with no modifiers or with both pre- and postnominal modifiers were excluded).

Group	Hard condition	Easy condition
Autism	0.31	0.13
Nonspectrum	0.26	0.18

### Exploratory comparisons linking child latencies and parent input characteristics

Because parental input was unscripted, we do not have balanced numbers of trials with different parent input features. Therefore, we cannot robustly analyze how specific parent input features might be associated with children’s processing. Nevertheless, to provide a basis for future work, we conducted some exploratory analyses. First, for the Easy condition, we compared latencies by whether the parent produced an unnecessary modifier (autistic group mean = 724 ms, *sd* = 969 ms; nonspectrum group mean = 318 ms, *sd* = 824 ms) or did not (autistic group mean = 1,093 ms, *sd* = 1,602 ms; nonspectrum group mean = 712 ms, *sd* = 1,727 ms). The number of data points in each of these cells is small and unequal, given that it depended on what parents chose to produce rather than our own manipulation (but recall that the use of unnecessary modifiers was relatively balanced; 51% for the autistic group and 60% for the nonspectrum group). Examining this pattern statistically, with latency as dependent measure, random effects of participant and trial, and fixed effects of group and modifier use and their interaction, we found no significant effects of group (*β* = 391.51, *p* = 0.13) or modifier use (*β* = −302.92, *p* = 0.10), and no significant interaction (*β* = −121.59, *p* = 0.73). This suggests that unnecessary modifiers did not substantially decrease processing efficiency.

We also examined latencies in the Easy and Hard conditions by whether the modifier, when present, occurred prenominally or postnominally. Again, this analysis is exploratory and limited by the number of data points per cell, which is uneven and very small in some cases. See Table 3. Like Arunachalam (2016), postnominal latencies were shorter than prenominal for the nonspectrum group in both conditions. However, this pattern did not hold for the autistic group. A model with latency as dependent measure, random effects of participant and trial, and fixed effects of group and modifier position and their interaction revealed a significant effect of group (*β* = 1096.53, *p* = 0.005), and of modifier position (*β* = 675.04, *p* = 0.048) and their interaction (*β* = 1861.72, *p* = 0.006). (We did not include condition in this model because both conditions showed the same pattern and we did not want to overfit the model.) We further explored the interaction with pairwise comparisons of the estimated marginal means, which revealed that the difference in latencies between modifier positions was significant for the autistic group (*t*(254.6) = −3.35, *p* = 0.005) but not the nonspectrum group (*t*(255.8) = 0.52, *p* = 0.95).

**Table 3:** Children’s mean latency to look at the target by group, condition, and modifier position (trials with no modifiers or with both pre- and postnominal modifiers were excluded).

Group	Condition	Modifier position	Number of data points	Mean latency, ms (sd)
Autism	Hard	Post	26	3,105.23 (6,148.79)
Autism	Hard	Pre	71	1,112.23 (1,795.23)
NS	Hard	Post	24	721.58 (962.93)
NS	Hard	Pre	63	994.32 (2,058.98)
Autism	Easy	Post	8	957.88 (883.31)
Autism	Easy	Pre	41	608.59 (910.67)
NS	Easy	Post	9	117.78 (346.08)
NS	Easy	Pre	41	388.17 (974.36)

This intriguing difference suggests that prenominal modifiers are more supportive than postnominal modifiers for comprehension for autistic children, contrary to what prior research has shown for nonspectrum children (and contrary to the trend, though not significant, for nonspectrum children in the current study); thus, they may benefit from different kinds of linguistic contexts for referential expressions than nonspectrum children.

## Discussion

To understand children’s language development, it is important to consider not only the language *input* they experience but also their *intake* from that input. The goals of the current study were to assess intake by measuring children’s comprehension of their parents’ language and to explore how parents might tune their speech to make the task of language comprehension easier for their child. We compared these features across groups, evaluating autistic and nonspectrum preschoolers, because for children on the spectrum, differences in understanding and making use of social cues, as well as less robust linguistic skill and slowed processing, may mean that they process less of the language input directed to them and/or may process language more slowly (e.g., Arunachalam and Luyster, 2016, 2018; Crandall et al., 2019). Although parents have been shown to tune their language input to their child’s expressive language, we suggested that they may be less attuned to their child’s real-time language *processing* skills, and therefore less able to tune their language input to support comprehension specifically.

Child–parent dyads played a game in which the parent verbally labelled one image from an array and the child’s task was to identify the correct image as quickly as possible. Children’s gaze was tracked while they participated. This paradigm allowed us to analyze children’s language processing and features of the parents’ language input in the same setting and in real time. Specifically, we examined features of parent language input when labeling the image, how quickly children looked to the correct referent, whether these two measures were related, and whether these patterns differed for autistic children as compared to nonspectrum children.

The primary findings were twofold. First, language processing speed in the autistic group was significantly slower than in the nonspectrum group. This finding is consistent with prior reports that language processing as measured in a variety of tasks, with and without eye-tracking, is on average slower in autistic children (e.g., Bavin et al., 2014; Ellis Weismer et al., 2016; Bavin and Baker, 2017; Marini et al., 2020). The current study further adds evidence that the difference in processing speed occurs even with unscripted speech from a speaker the child is very familiar with (as compared to pre-recorded speech streams typically used in eye-tracking studies).

Second, parents of autistic children did not significantly differ from parents of nonspectrum children on any of the measured language properties: speaking rate, use of unnecessary modifiers in their referential expressions (just over half the time), or position of those modifiers (which were primarily prenominal, but less so in the Hard condition than the Easy condition). The literature is mixed on whether parent language input differs to children on and off the spectrum (see, e.g., Bang et al., 2019; Woolard et al., 2021 for two recent reviews). The current study provides another finding to add to this literature from a specific situation—we suggest that when it comes to labelling a single image from an array in a finding game, parents of autistic children do not differ from parents of nonspectrum children in the rate of delivery or kind of language they use.

Taken together, the results suggest that although there is a group difference between nonspectrum and autistic children in language processing speed, the language input of parents of autistic children is not adapted *specifically* to support slower processing. This is not to say that parents are not aware of their child’s language abilities or that their input is not tuned in other ways; indeed, parents of children on the spectrum are very sensitive to their child’s language development and often delays in language are the parent’s first indicator that their child might need an autism evaluation (e.g., Chawarska and Volkmar, 2007; Garrido et al., 2018). Moreover, in the current study, parent input *was* adapted to the difficulty of the task across both nonspectrum and autistic groups. Specifically, in the harder condition in which there were competitor objects (the Hard condition), parents were more likely to place modifiers postnominally than in the easier condition in which there were no competitors from the same category (the Easy condition). Therefore, although parent speech is adapted to support children’s processing, it is not differentially so for nonspectrum versus autistic children.

Moreover, although we interpret this finding cautiously due to the nature of the experimental design—the number of relevant data points is constrained by what parents choose to produce—our exploratory analyses suggest children on the spectrum may benefit from different kinds of referential expressions than nonspectrum children. Specifically, while nonspectrum children showed a trend toward faster latencies with referential expressions that had postnominal rather than prenominal modifiers, and this is consistent with Arunachalam (2016), autistic children showed a significant difference in the opposite direction—they were faster with prenominal than postnominal modifiers.

In what follows we turn to how these findings contribute to the literature, theoretically and methodologically.

### Tuning of parental input

It is well established that the trajectory of a child’s development of language has many influences, including bidirectional influences between parent and child language, even beginning in infancy, and in autism as well as nonspectrum development (e.g., Huttenlocher et al., 2010; Bani Hani et al., 2013; Warlaumont et al., 2014; Wu and Gros-Louis, 2014; Yurovsky, 2018; Fusaroli et al., 2019; Choi et al., 2020; Odijk and Gillis, 2020; Quigley and Nixon, 2020; Leung et al., 2021). For example, in a longitudinal study, Fusaroli et al. (2019) found reciprocal associations between child and parent language in nonspectrum and autistic children. In addition to affirming previous findings that parent language features predict child language, they also documented the reverse, that children’s language features predicted parent language: children’s language at one visit predicted parent language at a subsequent visit. This work focused on classic measures of expressive language like MLU and word types/tokens. In a more recent study, Fusaroli et al. (2021) focusing on caregivers’ alignment (i.e., re-use of the child’s language in dyad conversations) showed that caregivers of autistic children tended to use less and different kinds of alignment in comparison to caregivers of nonspectrum children. Thus, parents appear to be tuned to their child’s expressive language abilities and to tune their own speech accordingly.

However, children’s expressive language is not the only domain to which parents are sensitive. For example, parents use infant-directed speech to infants and not older children, even before the infants use any expressive language at all (e.g., Fernald and Simon, 1984), and recent evidence shows that parents fine-tune how they label objects depending on their child’s knowledge about the object (Leung et al., 2021). Roy et al. (2009) found that the parents of one child produced a word in shorter utterances just *before* the child began to produce that word.

Despite these intriguing individual findings, what we know about how parents tune their speech to their child is limited, because most previous work showing reciprocal parent–child influences in both nonspectrum and autistic groups has focused on expressive language ability rather than language comprehension and processing. Chronological age is unlikely to be the sole factor, as is illustrated by evidence from autistic children and intellectual disability who show a gap between chronological age and expected language—these children receive input that is more tuned to their language level but not necessarily their chronological age (e.g., Bang et al., 2019). It is unsurprising that expressive language ability is an important factor that parents are sensitive to, because it is a salient part of how parents experience their child’s developing language ability. In the present study, we instead chose to focus on receptive language, aiming to tap into children’s intake of the input by investigating how quickly children comprehend the language produced by their parent. Although receptive and expressive language scores on standard assessments are strongly correlated in autistic children just as in nonspectrum children (Luyster et al., 2008), they are not perfectly correlated, and receptive language is often a domain of relative difficulty (e.g., Luyster et al., 2008; Hudry et al., 2010). It might be that parents are less sensitive to their child’s receptive abilities because they are less easily observed. Certainly, nonverbal communication, too, can provide a signal to parents of the child’s language level (e.g., Yoder and Warren, 1993), and parents respond accordingly, producing slightly more sophisticated language at each stage of the child’s development. However, the current study suggests that at least one aspect of receptive language ability, the speed with which children process language, is not a primary driver of parent tuning. Thus, the current study paves the way for examining which aspects of receptive language, over and above expressive language, parents are sensitive to.

### Methodology

In addition to the above implications, the current study also makes important methodological contributions, in two ways. First, in terms of measuring children’s language, our focus on language processing is important because language processing speed is related to children’s abilities to comprehend and learn from language in real time. Here, we used eye-tracking to measure processing, which provides an implicit measure of comprehension without requiring that the child execute motor actions or comply with instructions to speak—which may be difficult for autistic children (e.g., Kasari et al., 2013; Venker and Kover, 2015; Plesa Skwerer et al., 2016; Horvath and Arunachalam, 2019). This method offers strong potential for assessing receptive language in autism (Tager-Flusberg and Kasari, 2013).

Second, in terms of assessing parent–child interaction, prior work has mostly used either tightly controlled experimental designs to assess children’s language processing (e.g., Venker et al., 2013, 2019; Horvath and Arunachalam, 2019) or naturalistic observation of parent–child interaction to assess spontaneous parental input (see Bottema-Beutel and Kim, 2021). The relation between parents’ unscripted input and children’s intake *in the moment* had not previously been examined either in typical development or in autism (e.g., Bottema-Beutel and Kim, 2021). This work therefore moves beyond the pre-recorded stimuli used in most language processing experiments; we hope that it might generate future hypotheses that are testable within more controlled paradigms.

Critical to our study is Arunachalam’s (2016) paradigm, in which parents describe objects to children whose eye gaze is tracked, allowing analyses of both parental language and children’s processing in the same setting and in real-time. With respect to parents’ language, the game context offers some useful constraints over open-ended play sessions, because all participants are speaking about the same things and there are limited sources of variance in parents’ speech. This has some advantages for research aiming to look at very specific phenomena, as in the current study, where we looked at the referential expressions parents use to uniquely identify a referent in the context of distractors. With respect to children’s processing, this paradigm offers insight into how children process the language they are likely hearing in real life (i.e., from a familiar parent and in the way that person speaks given this kind of context). A recent word learning study finds that 2-year-olds with a higher likelihood of autism diagnosis process their parent’s voice effectively, allowing them to learn new words (van Rooijen et al., 2022). The current study, too, shows similar findings with slightly older children, in the context of unscripted speech.

### Limitations and future directions

Despite these advantages of the paradigm, it is also important to recognize several limitations of the current study. First, due to the nature of the methodology, parents’ speech in this study is inevitably less natural than everyday speech. In ongoing work, we are examining parents’ speech as they produce an unscripted narrative from a picture book (Shukla et al., 2022); we aim to be able to understand whether and how the patterns observed in the current study differ in more natural contexts. Second, relatedly, because of the unscripted nature of the task and the consequent variability in the language children are hearing, the findings about children’s processing are exploratory, particularly for features that were rarely produced by parents (e.g., postnominal modifiers) and for which our analyses are underpowered. Our sample, though comparable in size to several other experiments involving preschool-aged children on the spectrum (e.g., Naigles et al., 2011; Tenenbaum et al., 2017; Venker, 2019; Venker et al., 2019; Luyster and Arunachalam, 2020), is small, which is a limitation. We intentionally recruited from a relatively wide age range, intending to yield groups that were similar on language while acknowledging that they might differ greatly in chronological age; future studies, however, might benefit from concentrating on a narrower range.

Further, our sample does not fully capture the wide heterogeneity of the autism spectrum, and we expect that not all autistic children will have slow language processing. Moreover, our sample was limited by the fact that, like many experimental studies, we required families to visit the lab in order to participate, which might pose barriers related to family factors such as socioeconomic status and access to transportation as well as child factors such as interest in participating in activities outside the home.

We also recognize that parent–child interactions (e.g., Prevoo and Tamis-LeMonda, 2017) are culturally embedded, and that these patterns of dyadic engagement are likely to vary across samples that differ in communication traditions. However, it is also true that cross-cultural research attests to the capacity of adults to strategically modify their behaviors in order to improve communication efficacy (Agredo-Delgado et al., 2022).

Finally, another important limitation—perhaps one that especially highlights avenues for future work—is the fact that none of the participating parents reported having a diagnosis of autism themselves. (Note that although there has been research on parental traits within the broader autism phenotype, recent work has cautioned against treating this as the same as having a formal autism diagnosis; Sasson and Bottema-Beutel, 2021.) A particularly exciting area for future research involves parents with a diagnosis of autism. Many autistic adults report more successful communication and better social rapport with other autistic adults than with nonautistic adults (see, e.g., Bascom, 2012). Research on the double empathy problem (e.g., Milton, 2012) has not thus far focused on parent–child communication specifically. It would be particularly instructive to examine whether autistic parents use different communication strategies with their autistic children and whether such differences might sometimes lead to more successful communication and learning; we are pursuing these questions in ongoing research.

### Conclusion

We have framed children’s language acquisition as dependent not only on input, but crucially, also on intake—that is, how children process that input and the resulting linguistic representations they form. The current study supports prior work in documenting striking similarities between the input provided by parents of nonspectrum children and parents of autistic children, but we extend beyond prior work to show that children’s processing of this very input is slower in the autistic group than the nonspectrum group. Thus, the input is similar in many ways.

However, potential effects on language learning are cascading (e.g., Naigles and Tek, 2017; Arunachalam and Luyster, 2018), so slower processing may mean that autistic children could have less intake even with similar input. Although we examined only a brief interaction, in daily life, children who are slow language processors may be likely to miss opportunities to learn more language. Suppose that instead of simply naming an object, parents had continued their utterances to introduce something new, e.g., “there’s an open book… that’s on a desk.” A child who is slow to identify the open book will be less likely to have the opportunity to learn the meaning of “desk.” Prior work shows that difficulty processing the beginning of a sentence can indeed interfere with children’s abilities to learn new words that occur afterward (Fernald et al., 2008a;He et al., 2020; He et al., in prep). Therefore, less intake due to slower processing may have cascading effects on development of language skills, potentially contributing to explanations for the language difficulties seen in many autistic individuals throughout the lifespan.

## Data availability statement

The dataset analyzed for this study can be found at: 950 https://osf.io/fkhns/?view_only=3e5ae0ce585e40f6881f10fedf3901b0.

## Ethics statement

The studies involving human participants were reviewed and approved by Boston University Institutional Review Board. Written informed consent to participate in this study was provided by the participants’ legal guardian/next of kin.

## Author contributions

SA conceived of the study questions. SA and AH contributed to the design of the study and coded and analyzed the data. AH contributed to data collection. RL contributed to data interpretation. All authors contributed to the article and approved the submitted version.

## Funding

Research reported in this publication was supported by the National Institutes of Health under Award Number R01DC016592.

## Conflict of interest

RL is an author on the ADOS-2 and receives royalties from sales.

The remaining authors declare that the research was conducted in the absence of any commercial or financial relationships that could be construed as a potential conflict of interest.

## Publisher’s note

All claims expressed in this article are solely those of the authors and do not necessarily represent those of their affiliated organizations, or those of the publisher, the editors and the reviewers. Any product that may be evaluated in this article, or claim that may be made by its manufacturer, is not guaranteed or endorsed by the publisher.

## Footnotes

^1^Due to their ages, we did not directly ask the participants in the study whether they prefer to be referred to as "autistic" or "on the spectrum" (or something else), and so we use both interchangeably throughout the manuscript. We use "nonspectrum" to indicate children whose parents report that they are developing typically and do not have autism.

^2^Note that Arunachalam (2016) referred to these conditions as "Same" and "Different"; for ease of comprehension, we have changed them to "Hard" and "Easy," respectively.
