# Supplementary material for: Parental tuning of language input to autistic and nonspectrum children
Source: Front Psychol. 2022 Sep 23;13:954983. doi: 10.3389/fpsyg.2022.954983 (PMC9537044; doi:10.3389/fpsyg.2022.954983)
Supplement: Supplementary file 1 [file Table_1.docx]

Supplementary Materials

List of target items on all trials

| Target Item | Object competitor (Same condition only) |
| --- | --- |
| star (blue) | star (red) |
| bear (sleeping) | bear (awake) |
| book (open) | book (closed) |
| pumpkin (big) | pumpkin (small) |
| pencil (big/long) | pencil (small/short) |
| piano (small) | piano (big) |
| glass (small/short) | glass (big/tall) |
| girl (small/short) | girl (big/tall) |
| triangle | NA |
| apple | NA |
| boy | NA |
| house | NA |
| knife | NA |
| backpack | NA |
| hat | NA |
| umbrella | NA |
